# Dietary Fiber Intake Was Inversely Associated with All-Cause Mortality but Not with Cancer and Cardiovascular Disease Mortalities in the US

**DOI:** 10.3390/diseases13080272

**Published:** 2025-08-21

**Authors:** Zoha Akbar, Sundus Fituri, Zumin Shi, Vijay Ganji

**Affiliations:** 1 Department of Nutrition Sciences, College of Health Sciences, QU Health, Qatar University, Doha P.O. Box 2713, Qatar; za1404491@student.qu.edu.qa (Z.A.); sf1513326@student.qu.edu.qa (S.F.); zumin@qu.edu.qa (Z.S.); 2 Nutrition and Dietetics Program, School of Health and Human Sciences, Indiana University Indianapolis, Indianapolis, IN 46202, USA

**Keywords:** cancer, cardiovascular disease, dietary fiber, mortality, National Health and Nutrition Examination Survey, NHANES

## Abstract

Background: Evidence linking dietary fiber intake with cancer risk and mortality is equivocal. Objective: We investigated the relationship between dietary fiber intake and all-cause, cancer, and cardiovascular disease (CVD) mortalities in US adults ≥ 20 years. Methods: Data from the National Health and Nutrition Examination Surveys (NHANES) from 2003 to 2016 were used. Seven two-year cycles were concatenated into one analytic data file, NHANES 2003–2016 (*n* = 25,868; age ≥ 20 years). Dietary fiber intakes were collected from one 24-h dietary recall. Fiber intakes were categorized into quartiles. Mortality information was obtained from data linkage. To determine mortality, subjects were followed up for 6.4 years. Association between dietary fiber and mortality from all causes, cancer, and CVD was determined with multivariable-adjusted Cox proportional hazards models. Multivariate-adjusted Cox proportional hazard regression was used to generate mortality survival rates. Results: During the follow-up period, out of 2520 deaths, 561 and 511 deaths were from cancer and CVD, respectively. Dietary fiber intake was inversely associated with all-cause mortality [RR (95% CI), 0.67 (0.56–0.80); *p* ≤ 0.001]. No relationship was observed between fiber intake and cancer mortality [RR (95% CI), 0.8 (0.55–1.17); *p* = 0.51] and CVD mortality [RR (95% CI), 0.84 (0.53–1.33); *p* = 0.67]. Conclusions: In the US population, dietary fiber intake was associated with decreased all-cause mortality, but not with cancer and CVD mortality.

## 1. Introduction

The global burden of non-communicable diseases (NCDs) continues to pose a significant public health concern. As a leading cause of death, NCDs account for over 70% of mortality worldwide [1]. From 2007 to 2017, the number of premature deaths from NCDs alone increased by 22.7% [1]. Of all NCDs, cardiovascular disease (CVD) and cancer are the top two leading causes of death [2]. Given the preventable nature of many NCDs, the need for effective preventive strategies is critical for population health. Several modifiable lifestyle factors, such as smoking, physical inactivity, and consumption of an unhealthy diet, play a critical role in the prevention of NCDs [3].

The role of a healthy diet in the prevention of NCDs has long been investigated. Globally, the average consumption of healthy foods such as fruits, vegetables, wholegrains, nuts, and seeds does not meet optimal recommendations [4]. As a primary component of such foods, fiber has widely been recognized to be a key element of healthy diets recommended by several nutritional guidelines [5,6]. Dietary fiber is the non-digestible form of carbohydrates present in plant-based foods, and can be loosely classified as fermentable/soluble and non-fermentable/insoluble [7]. Overall dietary fiber intake in the US population is less than optimal.

Studies investigating the association of dietary fiber intake with all-cause, cancer, and CVD mortality have produced conflicting findings. In the Global Burden of Disease Study, low intakes of whole grains and fruits, which are primary sources of fiber, were found to be the most important dietary risk factors for mortality [8]. While some studies found no significant association between fiber intake and mortality [9,10], another found insoluble fiber to, in fact, increase mortality from colorectal cancer [11]. Moreover, other studies reported that higher intake of dietary fiber reduced the risk of multiple NCD mortalities, including those from cancer and CVD [12,13]. These positive outcomes could be attributed to several potential mechanisms through which fibers may act, which ultimately lead to improvements in blood pressure [14,15], serum cholesterol, especially LDL cholesterol [16,17,18], insulin sensitivity [19], glycemic control [20,21], weight loss [22,23], reduced fat digestion, and increased fat loss in stool, especially palmitic acid [24], oxidative stress [25,26], and chronic inflammation [27,28]. Over time, these effects could contribute to reduced mortality rates [13,29].

However, the evidence for the association between dietary fiber intake and mortality in the US population is not consistent [29]. Critical appraisal of meta-analysis studies found that the evidence linking dietary fiber intake with cancer mortality is weak [30]. A study by Park et al. [29] utilizing NIH-AARP cohort data reported no association between dietary fiber intake and all cancer mortality in women. Park et al. and Li et al. [31] observed an inverse association between grain fiber, but not with other fiber sources, and all-cause mortality. Moreover, many of the studies were conducted on populations such as post-menopausal women [11,32,33], older adults with hypertension [34], survivors of cancer [35], or patients with myocardial infarction [31]. Given these scenarios, the objective of the current study was to examine the association between intake of dietary fiber and mortality from all causes, CVD, and cancer in a large, nationally representative cohort of US adults.

## 2. Methods

### 2.1. Study Design and Sample Derivation

NHANES is a nationally representative study that began in the early 1960s and has since been conducted as a series of cross-sectional surveys designed to assess the health and nutritional status of the US population [36]. The NHANES survey design was a stratified, multistage probability sample. The survey was conducted on the noninstitutionalized civilian US population. Since 1999, NHANES surveys have been conducted as annual surveys, and data are released in two-year cycles in the public domain. The surveys use a complex, multi-stage, stratified, random sampling design and are implemented by the National Center for Health Statistics (NCHS), operating under the Centers for Disease Control and Prevention (CDC). Data on demographic characteristics, diet, and health are collected from personal interviews. Physical examinations and collection of blood and urine samples are conducted in the Mobile Examination Center (MEC). To generate reliable estimates, populations such as adolescents, low-income persons, persons > 60 years old, non-Hispanic blacks, and Hispanics/Mexicans were oversampled. Further details of the study are available to the public and have been described previously [37,38,39,40].

For this study, we used the data from NHANES 2003–2004, NHANES 2005–2006, NHANES 2007–2008, NHANES 2009–2010, NHANES 2011–2012, NHANES 2013–2014, and NHANES 2016–2016. These seven cycles of NHANES were combined into one analytic data file, NHANES 2003–2016.

The present analysis was conducted on adults ≥ 20 y old (*n* = 39,221). Participants with missing mortality data were excluded (*n* = 5719). We chose the ≥20-year-old population because cancer and CVD mortality are lower in children compared to the adult population. There was no restriction on upper age. Further, 7634 subjects without food intake data or those who reported total energy intake outside of pre-defined limits (800–4200 kcal/day for men; 500–3500 kcal for women) were excluded from the analyses [41,42] because these participants were considered outliers. Thus, the final analytic study sample was 25,868.

### 2.2. Outcome Variable: Mortality

The primary outcomes for this study were all-cause mortality, cancer mortality, and CVD mortality. Mortality data were obtained through linkage with death certificate records from the National Death Index (NDI), a centralized database maintained by the NCHS to provide vital status. All-cause mortality encompassed both identified and unidentified causes of death, whereas CVD and cancer-related mortalities were defined using the International Classification of Diseases coding. The length of follow-up was calculated from the date of the survey participation to the date of the last follow-up or death, whichever came first.

### 2.3. Exposure Variable: Dietary Fiber Intake

The assessment of fiber intake was based on the NHANES dietary interview component called What We Eat in America (WWEIA), conducted as part of a survey in collaboration with the US Department of Agriculture (USDA) [43]. Details of this component have been published elsewhere [44]. In brief, the dietary interview component comprised two 24-h recalls using the USDA’s Automated Multiple-Pass Method for each NHANES participant [45]. The first dietary recall was administered face-to-face by trained interviewers in the Mobile Examination Center (MEC), while the second was conducted via telephone in the following 3 to 10 days. To maintain consistency in the dietary information obtained, only the first 24-h recall was used in the present analysis. The sum of the fiber content of each food/beverage reported in the 24-h recalls was calculated using the USDA’s Food and Nutrient Database for Dietary Studies (FNDDS) [46]. The FNDDS is a food composition database based on the USDA’s National Nutrient Database for Standard Reference that is used to assign codes to the various foods and beverages reportedly consumed by the participants of NHANES. The food composition for this database is obtained from various sources, including scientific literature, information supplied by food manufacturers, and chemical analyses commissioned by the USDA. The FNDDS is consistently updated by the USDA every two years to reflect changes in dietary consumption patterns and the marketplace during each survey period.

### 2.4. Covariates

In the present analysis, age, sex, race, ethnicity, energy intake, fat intake, physical activity, education, income, smoking, alcohol, body mass index (BMI), and hypertension were treated as covariates. All these covariates were considered as confounding variables in the data analysis. Although NHANES collected data from minority groups other than non-Hispanic black and Hispanic/Mexican Americans, the sample sizes for these groups were small. Therefore, we created a category called “Others”, which included all minority groups except non-Hispanic Black and Hispanic/Mexican Americans. The covariates were selected based on known possible confounders of the fiber–mortality relationship. Physical activity was determined using the Global Physical Activity Questionnaire, a validated instrument designed to collect data on three domains, namely activity related to work, travel, and recreation [47]. These were then recoded into three categories of Metabolic Equivalent of Task (MET) minutes per week (<600, 600–1200, and >1200), indicating their intensity levels as either low, medium, or high. Education level was evaluated by the highest degree achieved or the highest grade of school completed (<11th grade, high school graduate/GED or equivalent, some college or AA degree, college graduate, or above). The classification of income was based on the Poverty Income Ratio (<1.3, 1.3–3.5, and >3.5) derived from the division of family income by the poverty threshold. Smoking status was categorized as current, former, or never. Hypertension was recoded as one variable defined by either self-reported diagnosis or systolic blood pressure ≥ 140 mmHg and/or diastolic blood pressure ≥ 90 mmHg.

### 2.5. Data Analysis

Dietary fiber intakes were categorized into quartiles. For the description of the sample characteristics, differences between groups were compared using the chi-square test for categorical variables and ANOVA for continuous variables. Adjusted hazard ratios (HRs) were determined for all-cause mortality, cancer mortality, and CVD mortality through multivariable Cox proportional regression using the lowest category of fiber intake as a reference. The first model was adjusted for age, sex, and energy intake. The second model was adjusted for age, sex, energy intake, fat intake, physical activity, education, income, smoking status, and alcohol consumption. The third model was adjusted for age, sex, energy intake, fat intake, physical activity, education, income, smoking status, alcohol consumption, BMI, and hypertension. Potential modification of the association between fiber intake and all-cause mortality by various factors (race, gender, age, income, and education) was tested in subgroup analyses through the addition of interaction terms in the regression models. Cox proportional hazard regression analysis was performed to generate the survival rates by the level of fiber intake after adjusting for age, sex, energy intake, fat intake, physical activity, education, income, smoking status, alcohol consumption, BMI, and hypertension. Sampling weights were used in the multivariable analyses to account for the complex survey design of the NHANES. Statistical significance was considered when *p*-values were <0.05. All the analyses were performed using STATA 18.0 software.

## 3. Results

### 3.1. Sample Characteristics

A total of 25,868 adults were included in this study, with a mean follow-up duration of 6.5 years. Thus, the total follow-up person-years were 168,759. Table 1 presents the sample characteristics stratified by quartiles of fiber intake. Participants in the highest quartile of fiber intake were more likely to be men and non-smokers, and they reported higher energy intake and levels of physical activity. Furthermore, individuals in the highest quartile tended to have a higher socioeconomic status, as indicated by a greater family income-to-poverty ratio.

### 3.2. Associations Between Fiber Intake and Mortality

During the 6.5-year follow-up period, a total of 2520 deaths were reported, of which 561 and 511 deaths were attributed to cancer and CVD, respectively (Table 2). The rates (per 1000) of all-cause, cancer, and CVD mortality were lower in the highest quartile of fiber intake compared to the lowest quartile at 6.82 vs. 12.61, 1.73 vs. 2.47, and 1.27 vs. 2.42, respectively.

In the fully adjusted multivariate Cox regression model, higher dietary fiber intake was significantly associated with a decreased risk of all-cause mortality [RR (95% CI), 0.67 (0.56–0.80); *p* ≤ 0.001] (Table 2). In the age, sex, and energy intake adjusted model, higher quartile intakes compared to lower quartile intakes of dietary fiber were associated with reduced risk of cancer and CVD mortality. However, the associations were no longer significant in the fully adjusted model. Similarly, there was no association between dietary fiber intake and all cancer mortality (Table 2).

Subgroup analyses were conducted to assess whether associations between dietary fiber intake and all-cause mortality were modified by sociodemographic and lifestyle factors. No significant interactions were observed between dietary fiber intake and the covariates examined in relation to all-cause mortality (Table 3).

**Figure 1:**
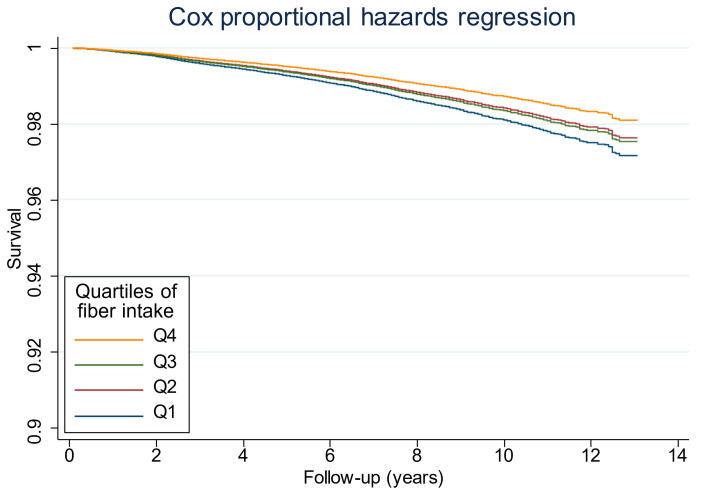
Association between fiber intake and survival estimates in the National Health and Nutrition Examination Survey, 2003–2016. Dietary fiber intake quartiles: Q1, <10.5 g, Q2, 10.5 g to <14.8 g; Q3, 14.8 g to 20.6 g; Q4, ≥20.6 g. Multivariable adjusted survival estimates were calculated using Cox proportional hazards regression analysis. Analysis was adjusted for age, sex, energy intake, fat intake, physical activity, education, income–poverty ratio, smoking, alcohol drinking, BMI, and hypertension. Persons in the 4th quartile of fiber intake had a significantly higher survivor probability compared to those in the 1st quartile of fiber intake.

Furthermore, Cox proportional hazard analysis curves showed a significant difference in the probability of survival. Participants in the highest quartile of fiber intake had the highest probability of survival as compared with those in the lowest quartile of fiber intake. However, the survival curves overlapped between fiber intake quartiles 2 and 3, revealing higher survival rates in participants with higher fiber intake compared to those with lower fiber intake (Figure 1).

## 4. Discussion

In this large, nationally representative sample of the US population, we found a decreased risk of all-cause mortality with increased dietary fiber intake. Also, we found that high fiber intake is associated with higher survival rates. However, no significant associations were found between dietary fiber intake and mortality from cancer or CVD.

Our results are consistent with a study reporting an inverse relationship between dietary fiber intake and all-cause mortality [48]. Cox proportional survival curves also lend support to these findings. Survival curves showed that the survival probability is higher in those persons who consumed the highest amount (4th quartile) of dietary fiber compared to those who consumed the lowest amount (1st quartile). Yao et al. [48] investigated a dose–response relationship utilizing the data from 14 studies (*n* = 1,367,285) and reported an overall RR of 0.90 (95% CI: 0.86–0.93; *I*^2^ = 86.1%, *P_heterogeneity_* < 0.001) for every 10 g/day of dietary fiber intake increment. However, the lack of association with cancer-specific and CVD-specific mortality contrasts with other studies that reported reduced risk of cancer and CVD mortality at higher insoluble fiber intakes [49]. They reported a significant reduction in mortality from cancer or CVD with only insoluble fiber (HR for quintile 5 vs. quintile 1: 0.65; 95% CI: 0.45, 0.94; *p* = 0.02). Interestingly, in contrast to our findings, they did not find any association with total dietary fiber intake [49]. A study on the Korean population found an inverse association between dietary fiber intake and all-cause and CVD mortality [50]. In this current study, in a limited adjusted model, an inverse association between increased fiber consumption and CVD and cancer mortality was observed, which disappeared after adjusting for additional confounders such as fat intake, physical activity, and other lifestyle factors, highlighting the multifactorial etiology of cancer and CVD-related mortality. The differences in findings between our study and theirs are more likely due to differences in subject characteristics, dietary intake measurement, and model adjustments.

In a meta-analysis, dietary fiber intake ranging from 25 to 29 g provided the most significant reduction (about 15 to 30%) in all-cause and CVD mortality risk, while it was indicated that consuming over 30 g of dietary fiber per day could provide further health advantages [51]. A meta-analysis by Yao et al. [48] found an inverse association of cancer mortality only with cereal fiber, while in a cohort of US adults, there was no notable association between fiber consumed for breakfast and lunch and the risks of cancer and CVD mortality, but lower risks of cancer mortality were observed in participants in the highest quintile of fiber consumed for dinner [52]. The lack of association between fiber intake and CVD and cancer mortality in the present study could also be partially attributed to fiber types and meal timing. For an instance, soluble fiber from oats or psyllium is known to reduce the risk for heart diseases by lowering blood total cholesterol and LDL cholesterol [16,53], while insoluble fiber from legumes is known to reduce the risk of cancer, especially colorectal cancer, by reducing the transit time of the stool through the gut [54]. It is possible that individuals may have consumed one type of fiber over the other. Although specific fibers have specific health benefits, these health benefits did not translate into decreased mortality related to cancer or CVD in our study. We were unable to determine the specific dietary fiber composition of individuals because the NHANES did not collect this specific data. Again, we were unable to determine the fiber consumption by meal because this data is not collected by the NHANES.

Furthermore, some studies have reported a more robust association of fiber intake with non-CVD and non-cancer inflammatory diseases [55,56]. One potential mechanism underlying the inverse association between dietary fiber intake and all-cause mortality observed in this study may be explained by short-chain fatty acid (SCFA) production, which occurs via the fermentation of soluble dietary fiber by the intestinal microbiota [57,58]. SCFAs play a role in the maintenance of the gut barrier and protection against inflammation and gut microbiota dysbiosis [59,60]. Additionally, these SCFAs have been reported to reduce the endogenous hepatic cholesterol synthesis by inhibiting HMG-CoA reductase [61]. Consumption of dietary fiber is linked to decreased concentrations of inflammatory markers such as C-reactive protein and tumor necrosis factor-α receptor-2 [62,63]. Additionally, dietary fiber intake increases satiety through delayed gastric emptying [64], aiding in weight management and reducing glycemic response to food [65,66]. These have all been well-documented to reduce the risk of several chronic diseases. In this study, the reduced overall mortality risk with increased dietary fiber intake is likely due to its impact on non-cancer or non-CVD diseases.

This study has strengths such as its large sample size, long follow-up duration, and use of nationally representative data. The analysis was adjusted for several potential confounders, enhancing the robustness of the findings. However, there are some limitations. We did not analyze the associations of different types of fiber with all-cause, cancer, and CVD mortality due lack of this data in the NHANES database. Furthermore, dietary intake was based on one 24-h food recall with no additional diet recalls collected face-to-face during follow-up. Dietary intake data from one 24-h food recall may not reflect habitual consumption. Also, subjects may not be able to recollect all the foods/beverages that they have consumed in the last 24 h. Therefore, actual dietary intakes may be underestimated with 24-h food recalls, leading to the underestimation of dietary fiber intake. We do not know the impact of this on the outcomes of this study. Although we have adjusted the analysis for several potential confounding variables, residual confounding may still be present.

In conclusion, high dietary fiber consumption was associated with reduced risk of all-cause mortality in the US population. This reduced risk of all-cause mortality is not due to a reduction in cancer or CVD mortality. No significant associations were observed between fiber intake and CVD and cancer-related mortality. Further research is needed to elucidate the underlying mechanisms, such as types and timing of fiber intake, and clarify the inconsistencies in the association between dietary fiber intake and mortality. Further, the relationship between natural dietary fiber and fiber added during processing and mortality should be studied.

Our study findings highlight the importance of dietary fiber as part of a healthy diet. Based on the national consumption studies, 95% of the US population does not meet the recommended intake of dietary fiber. The Institute of Medicine recommends 19 to 38 g/day of dietary fiber [67]. With increased popularity of gluten-free diets in the US is further contributing to lower intakes of dietary fiber. A summit on Food and Fiber proposed better communication strategies for practitioners that would identify barriers related to fiber and help promote its intake [68].

## Figures and Tables

**Table 1:** Sample characteristics by quartiles of dietary fiber intake in National Health and Nutrition Examination Survey, 2003–2016 ^1,2^.

	Q1	Q2	Q3	Q4	*p*-Value
	*n* = 6474	*n* = 6484	*n* = 6476	*n* = 6434	
Fiber intake, g/d	7.6 ± 2	12.6 ± 1.2	17.4 ± 1.6	27.7 ± 7.3	<0.001
Fat intake, g/d	57.2 ± 25.4	70.4 ± 29	79.5 ± 32.3	90 ± 35.9	<0.001
Energy intake, kcal/d	1527 ± 551	1856 ± 603	2109 ± 639	2460 ± 695	<0.001
Age, y	49.2 ±18.5	49.8 ±18.4	50.9 ± 18.1	50.1 ± 17.2	<0.001
Sex					<0.001
Men, *n* (%)	2458 (38)	2689 (41.5)	3162 (48.8)	3828 (59.5)	
Women, *n* (%)	4016 (62)	3795 (58.5)	3314 (51.2)	2606 (40.5)	
Ethnicity					<0.001
Non-Hispanic White, *n* (%)	2993 (46.2)	3107 (47.9)	3295 (50.9)	3065 (47.6)	
Non-Hispanic Black, *n* (%)	1912 (29.5)	1501 (23.1)	1136 (17.5)	773 (12)	
Hispanic/Mexican American, *n* (%)	681 (10.5)	898 (13.8)	2 (16.2)	1527 (23.7)	
Others, *n* (%	888 (13.7)	978 (15.1)	993 (15.3)	1069 (16.6)	
Smoking					<0.001
Never, *n* (%)	3067 (47.4)	3536 (54.6)	3689 (57)	3767 (58.6)	
Former, *n* (%)	1400 (21.6)	1597 (24.6)	1760 (27.2)	1879 (29.2)	
Current smoker, *n* (%)	2004 (31)	1347 (20.8)	1026 (15.8)	786 (12.2)	
Alcohol drinking					<0.001
No, *n* (%)	1348 (20.8)	1257 (19.4)	1199 (18.5)	1118 (17.4)	
Yes, *n* (%)	3813 (58.9)	3993 (61.6)	4072 (62.9)	4155 (64.6)	
Missing, *n* (%)	1313 (20.3)	1234 (19)	1205 (18.6)	1161 (18)	
Season					<0.001
Winter, *n* (%)	2914 (45)	2943 (45.4)	2933 (45.3)	3162 (49.1)	
Summer, *n* (%)	3560 (55)	3541 (54.6)	3543 (54.7)	3272 (50.9)	
BMI, kg/m^2^	29.5 ± 7.3	29.6 ± 7	28.8 ± 6.5	28.3 ± 6.2	<0.001
Leisure time physical activity (METs min/wk)					<0.001
<600, *n* (%)	3240 (50)	2998 (46.2)	2698 (41.7)	2183 (33.9)	
600–1200, *n* (%)	696 (10.8)	851 (13.1)	840 (13)	844 (13.1)	
≥1200, *n* (%)	2538 (39.2)	2635 (40.6)	2938 (45.4)	3407 (53)	
Ratio of family income to poverty					<0.001
<1.30, *n* (%)	2352 (36.3)	105 (27.8)	1608 (24.8)	1519 (23.6)	
1.3–3.5, *n* (%)	2310 (35.7)	2397 (37)	2247 (34.7)	2100 (32.6)	
>3.5, *n* (%)	1381 (21.3)	1845 (28.5)	2140 (33)	2370 (36.8)	
Missing, *n* (%)	431 (6.7)	437 (6.7)	481 (7.4)	445 (6.9)	
Hypertension, *n* (%)	2461 (39.5)	2404 (38.5)	2351 (37.5)	2077 (33.2)	<0.001

^1^ Data are presented as mean ± SD for continuous measures, and *n* (%) for categorical measures. ^2^ Dietary fiber intake quartiles: Q1, <10.5 g, Q2, 10.5 g to <14.8 g; Q3, 14.8 g to 20.6 g; Q4, ≥20.6 g.

**Table 2:** Association between dietary fiber intake and all-cause mortality, cancer mortality, and CVD mortality in the National Health and Nutrition Examination Survey, 2003–2016 ^1^.

	Q1	Q2	Q3	Q4	*p* for Trend
All-cause mortality					
Cases	768	664	646	442	
Rate (per 1000)	12.61	9.93	9.87	6.82	
Model A ^2^	1	0.71 (0.62–0.82)	0.66 (0.58–0.75)	0.47 (0.4–0.55)	<0.001
Model B ^3^	1	0.83 (0.72–0.95)	0.83 (0.72–0.95)	0.65 (0.55–0.77)	<0.001
Model C ^4^	1	0.83 (0.72–0.96)	0.87 (0.75–1.01)	0.67 (0.56–0.8)	<0.001
Cancer mortality					
Cases	160	140	152	109	
Rate (per 1000)	2.47	2.09	2.46	1.73	
Model A ^2^	1	0.75 (0.56–1)	0.8 (0.59–1.07)	0.55 (0.39–0.78)	0.003
Model B ^3^	1	0.89 (0.67–1.18)	1.05 (0.77–1.42)	0.81 (0.56–1.17)	0.51
Model C ^4^	1	0.87 (0.65–1.17)	1.04 (0.76–1.43)	0.80 (0.55–1.17)	0.51
CVD mortality					
Cases	162	130	132	87	
Rate (per 1000)	2.42	1.91	1.99	1.27	
Model A ^2^	1	0.77 (0.57–1.02)	0.78 (0.57–1.06)	0.56 (0.39–0.79)	0.002
Model B ^3^	1	0.89 (0.66–1.21)	0.97 (0.68–1.4)	0.79 (0.53–1.19)	0.42
Model C ^4^	1	0.9 (0.64–1.26)	1.03 (0.70–1.5)	0.84 (0.53–1.33)	0.67

^1^ Dietary fiber intake quartiles: Q1, <10.5 g, Q2, 10.5 g to <14.8 g; Q3, 14.8 g to 20.6 g; Q4, ≥20.6 g. ^2^ Adjusted for age, sex, and energy intake. Values are RR and their 95% CI. ^3^ Adjusted for age, sex, energy intake, fat intake, physical activity, education, income–poverty ratio, smoking, and alcohol drinking. Values are RR and their 95% CI. ^4^ Adjusted for age, sex, energy intake, fat intake, physical activity, education, income–poverty ratio, smoking, alcohol drinking, BMI, and hypertension. Values are RR and their 95% CI.

**Table 3:** Subgroup analyses for the association between dietary fiber intake and all-cause mortality in the National Health and Nutrition Examination Survey, 2003–2016 ^1,2^.

Quartiles of Fiber Intake									
	Q1	Q2		Q3		Q4		*p* Trend	*p* Interaction
Ethnicity									0.071
Non-Hispanic White	1	0.8	(0.68–0.94)	0.82	(0.69–0.98)	0.64	(0.51–0.8)	≤0.001	
Non-Hispanic Black	1	0.99	(0.76–1.28)	1.04	(0.75–1.43)	0.80	(0.52–1.22)	0.58	
Hispanic/Mexican American	1	1.01	(0.67–1.52)	0.9	(0.56–1.47)	0.79	(0.52–1.2)	0.22	
Others	1	0.84	(0.47–1.48)	1.2	(0.74–1.95)	0.59	(0.29–0.2)	0.36	
Sex									0.71
Men	1	0.8	(0.65–0.98)	0.8	(0.65–0.98)	0.65	(0.51–0.83)	0.001	
Women	1	0.87	(0.71–1.06)	0.99	(0.79–1.24)	0.69	(0.52–0.92)	0.1	
Age group									0.28
20~59	1	0.73	(0.53–1.01)	0.93	(0.67–1.28)	0.61	(0.41–0.93)	0.08	
≥60	1	0.88	(0.76–1.02)	0.84	(0.72–0.99)	0.70	(0.57–0.85)	0.001	
Ratio of family income to poverty									0.17
<1.30	1	0.97	(0.74–1.26)	1.03	(0.77–1.37)	0.92	(0.68–1.24)	0.77	
1.3–3.5	1	0.75	(0.62–0.91)	0.72	(0.57–0.89)	0.53	(0.41–0.7)	<0.001	
>3.5	1	0.87	(0.63–1.22)	0.86	(0.62–1.19)	0.69	(0.44–1.11)	0.14	
Missing	1	0.55	(0.3–0.99)	1.37	(0.71–2.67)	0.58	(0.23–1.48)	0.99	
Education									0.13
<11 grade	1	0.82	(0.64–1.03)	1.13	(0.9–1.43)	0.84	(0.62–1.15)	0.85	
High school	1	0.88	(0.68–1.15)	0.72	(0.52–1)	0.60	(0.42–0.86)	0.005	
Some college	1	0.73	(0.59–0.90)	0.68	(0.52–0.88)	0.56	(0.38–0.83)	0.003	
Higher than college	1	0.83	(0.5–1.38)	0.94	(0.6–1.48)	0.68	(0.4–1.18)	0.24	

^1^ Adjusted for the same covariates as Model C in Table 2. Values are RR and their 95% CI. ^2^ Dietary fiber intake quartiles: Q1, <10.5 g, Q2, 10.5 g to <14.8 g; Q3, 14.8 g to 20.6 g; Q4, ≥20.6 g.

## Data Availability

Data are available upon request from Z.S.
